# Excessive Signal Transduction of Gain-of-Function Variants of the Calcium-Sensing Receptor (CaSR) Are Associated with Increased ER to Cytosol Calcium Gradient

**DOI:** 10.1371/journal.pone.0079113

**Published:** 2013-11-14

**Authors:** Marianna Ranieri, Grazia Tamma, Annarita Di Mise, Giuseppe Vezzoli, Laura Soldati, Maria Svelto, Giovanna Valenti

**Affiliations:** 1 Department of Biosciences, Biotechnologies and Biopharmaceutics, University of Bari, Bari, Italy; 2 Nephrology and Dialysis Unit, San Raffaele Hospital, Scientific Institute, Milan, Italy; 3 Department of Health Sciences, University of Milan, Milan, Italy; 4 Centre of Excellence Genomic and Proteomics GEBCA, University of Bari, Bari, Italy; University of Miami School of Medicine, United States of America

## Abstract

In humans, gain-of-function mutations of the calcium-sensing receptor (CASR) gene are the cause of autosomal dominant hypocalcemia or type 5 Bartter syndrome characterized by an abnormality of calcium metabolism with low parathyroid hormone levels and excessive renal calcium excretion. Functional characterization of CaSR activating variants has been so far limited at demonstrating an increased sensitivity to external calcium leading to lower Ca-EC50. Here we combine high resolution fluorescence based techniques and provide evidence that for the efficiency of calcium signaling system, cells expressing gain-of-function variants of CaSR monitor cytosolic and ER calcium levels increasing the expression of the Sarco-Endoplasmic Reticulum Calcium-ATPase (SERCA) and reducing expression of Plasma Membrane Calcium-ATPase (PMCA). Wild-type CaSR (hCaSR-wt) and its gain-of-function (hCaSR-R990G; hCaSR-N124K) variants were transiently transfected in HEK-293 cells. Basal intracellular calcium concentration was significantly lower in cells expressing hCaSR-wt and its gain of function variants compared to mock. In line, FRET studies using the D1ER probe, which detects [Ca2+]ER directly, demonstrated significantly higher calcium accumulation in cells expressing the gain of function CaSR variants compared to hCaSR-wt. Consistently, cells expressing activating CaSR variants showed a significant increase in SERCA activity and expression and a reduced PMCA expression. This combined parallel regulation in protein expression increases the ER to cytosol calcium gradient explaining the higher sensitivity of CaSR gain-of-function variants to external calcium. This control principle provides a general explanation of how cells reliably connect (and exacerbate) receptor inputs to cell function.

## Introduction

The extracellular calcium-sensing GPCR (CaSR) belongs to the C family of the G-protein-coupled receptors GPCR, expressed primarily, but not exclusively, in parathyroid glands and kidney [1], [2]. The CaSR senses changes in extracellular calcium concentrations and regulates parathyroid hormone (PTH) secretion and renal tubular calcium reabsorption to maintain serum calcium levels within the normal range [3], [4], [5], [6], [7].

Ligand binding by the CaSR results in conformational changes of the intracellular loops, G protein-dependent stimulation of phospholipase C causing an accumulation of inositol 1,4,5-trisphosphate and rapid release of calcium ions from intracellular stores. The increase in intracellular calcium results in activation of protein kinase C and CaSR also activates the mitogen-activated protein kinase (MAPK) pathway [2], [8].

Mutations in CaSR coding gene have been associated with human diseases [9]. Loss-of-function CaSR mutations result in familial (benign) hypocalciuric hypercalcemia (FBHH), and neonatal severe primary hyperparathyroidism (NSHPT), characterized by resistance to the normal inhibition of PTH secretion by the hormone agonist, extracellular calcium [10], [11],[12].

Conversely, CaSR gain-of-function mutations cause autosomal dominant hypocalcemia (ADH) or type 5 Bartter syndrome, due to activation of the receptor at concentrations of serum calcium below physiological levels leading to abnormal inhibition of PTH secretion [13], [14], [15]. ADH patients display low serum calcium, normal or low PTH levels, sometimes associated with hypercalciuria and a Bartter-like syndrome, which predisposes those patients to nephrocalcinosis [13], [14], [15]. Another serious complication associated with activating CaSR mutations is a defect in bone mineralization [16] highlighting the importance of this receptor in skeletal function as well [17]. So far, more than 50 activating mutations of the CaSR have been identified to cause ADH (http://www.casrdb.mcgill.ca).

Similar to patients with ADH, mouse models for an activating CaSR mutation display hypocalcemia, hyperphosphatemia and inappropriately reduced levels of plasma PTH [18]. Transient expression of wild-type and mutant CaSRs in human embryonic kidney (HEK) cells demonstrated that the mutation resulted in a gain-of-function of the CaSR, which had a significantly lower EC_50_ (‘left shift’) [19], [20]. In addition to the activating mutation causing ADH it has been reported that R990G polymorphism of the CaSR also results in a gain-of-function of the receptor and increased susceptibility to primary hypercalciuria [19], [21].

Hypocalcemia in ADH patients is frequently treated with calcium and vitamin D; however, this treatment can lead to exacerbation of hypercalciuria, resulting in nephrocalcinosis, nephrolithiasis and chronic renal failure [10], [22]. Alternatively, calcilytics which lower the sensitivity of the CaSR to external calcium might offer a novel treatment option in patients with ADH, although it is not known whether calcilytics would be effective on mutated CaSR in ADH patients [23].

Clearly the best therapy for these diseases would be to directly correct the underlining molecular defect of the receptor. However, besides the reported increased sensitivity of the CaSR to external calcium leading to lower Ca-EC_50_, little is known about the molecular basis of gain-of-function variants of the CaSR.

Stimulation of CaSR elicits calcium mobilization from intracellular stores rather then extracellular calcium influx [24]. To understand the mechanism of CaSR mediating signaling and its alterations in gain-of-function CaSR expressing cells, is therefore essential to study the dynamics of intracellular calcium mobilization ranging from transient and oscillatory responses to sustained responses.

On the other hand, to maintain organellar calcium stores and the appropriate concentration gradients across cell membranes, mammalian tissues employ a large number of calcium-transporting ATPases belonging to three subfamilies of the P-type superfamily of ion-transport ATPases. These are the Secretory Pathway Calcium-ATPases (SPCAs) [25], the Sarco-Endoplasmic Calcium-ATPases (SERCAs) [26] and the Plasma Membrane Calcium-ATPases (PMCAs) [27]. Together, these pumps establish and maintain the calcium gradients that are essential for physiological processes in which calcium plays a prominent role. While the functions of the mammalian SPCA isoforms are not well understood it is clear that SERCAs and PMCAs play major roles in calcium signaling [28].

We report here that a complex parallel adaptive feedback can explain the molecular basis of gain-of-function variants of the CaSR. This involves a tight control of cytosolic and ER calcium levels and parallel regulation of SERCA expression in the ER and of PMCA at the plasma membrane.

## Results

### Expression and Localization of CaSR in HEK-293 Cells

**Figure 1:**
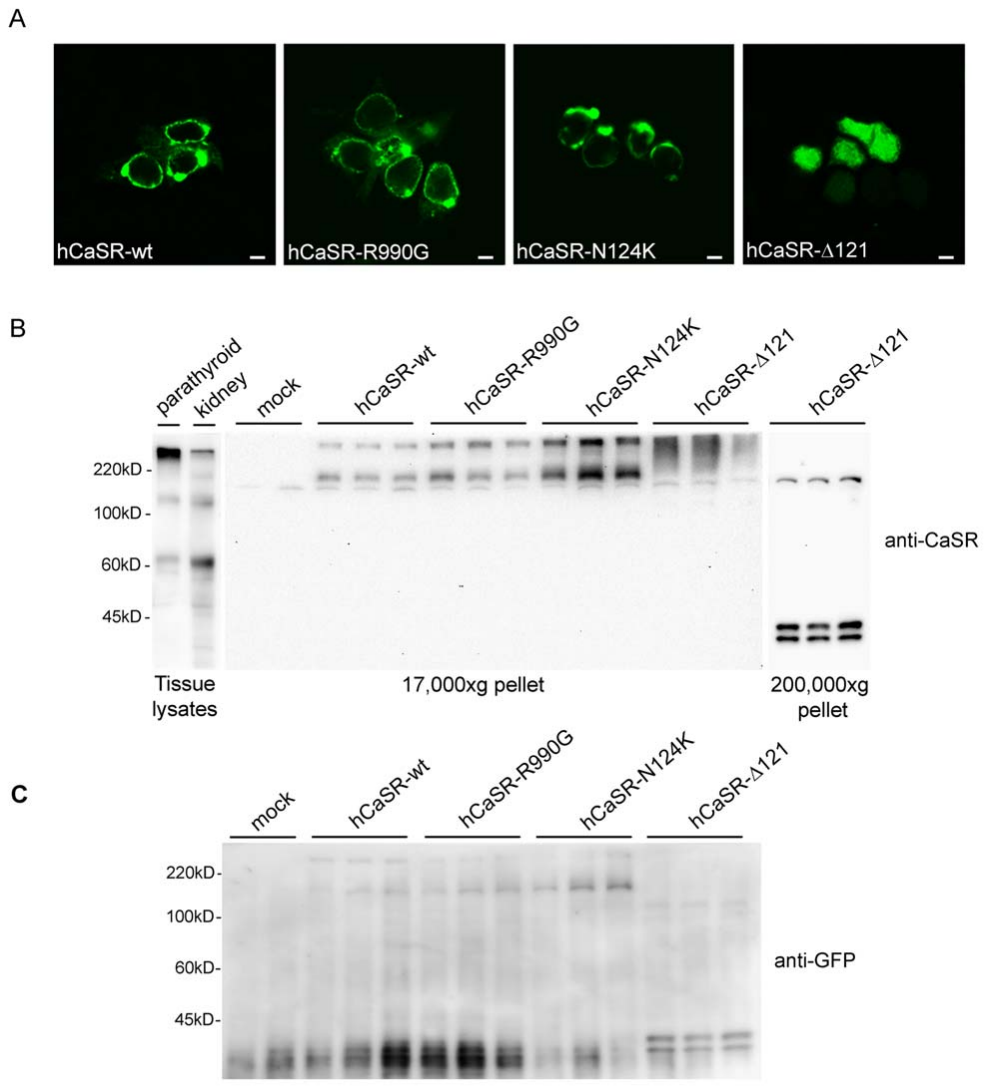
Expression and localization of Calcium Sensing Receptors (CaSR) in HEK-293 cells. (A) Confocal microscopy showing cellular localization of hCaSR-wt and the gain-of-function variants hCaSR-R990G and hCaSR-N124K. hCaSR-wt as well as its active variants, were mainly expressed at the plasma membrane. In contrast, hCaSR-Δ121, corresponding to a loss-of-function truncated protein, localized intracellularly. The scale bar corresponds to 5 µm. (B) Western blotting analysis. Equal amounts of proteins (60 µg) isolated from tissue lysates (parathyroid and rat kidney) or from cell 17,000×g pellet were immunoblotted and probed with monoclonal antibodies against CaSR (N-terminal, 1∶800). In lysates isolated from rat parathyroid and rat kidneys a band at approximately 135 kDa and an aggregate at higher molecular weight was stained. An immunoreactive band of ≈170 kDa, a band greater than 220 kDa marker and likely corresponding to higher molecular mass forms of the receptor were detected only in cells transfected with hCaSR-wt, hCaSR-R990G and hCaSR-N124K. The higher molecular weight observed with respect to native tissues is due to the presence of the GFP tag. In cells transfected with hCaSR-Δ121, the antibody stained bands at approximately 40 kDa in a 200,000×g pellet consistent with a prominent intracellular distribution. (C) Cell extracts from cells transfected with hCaSR-wt, hCaSR-R990G, hCaSR-N124K and hCaSR-Δ121 were immunoblotted and probed with a monoclonal anti-GFP antibody (1∶5000). Anti-GFP antibody stained bands at the same molecular weight as those revealed by anti CaSR antibody confirming the specificity of the revealed bands.

HEK-293 cells were transiently transfected with vectors encoding for the fused CaSRs with GFP, as described in Methods. Confocal analysis revealed that hCaSR-wt as well as its gain-of-function variants, hCaSR-R990G [19], [29] and hCaSR-N124K [20], were mainly expressed at the plasma membrane displaying comparable expression efficiency in HEK-293 cells. In contrast, hCaSR-Δ121, corresponding to a loss-of-function truncated protein [30], localized intracellularly (Fig. 1A). By immunoblotting, a monoclonal antibody recognizing the extracellular N-terminus of CaSR (amino-acids 19–29) stained a band at approximately 135 kDa and an aggregate at higher molecular weight in lysates isolated from rat parathyroids and rat kidneys (Fig. 1B). In a membrane-enriched fraction (17,000×g pellet) of transfected cells specific bands corresponding to hCaSR-wt, hCaSR-R990G and hCaSR-N124K were detected approximately at 170 kDa. The higher molecular weight observed with respect to native tissues is due to the presence of the GFP tag. In addition, higher molecular mass forms, presumably dimers, were seen for all receptor wild type and gain-of-function variants. The exogenous expression of the truncated non-functional hCaSR-Δ121 protein was detected only in the intracellular enriched fraction (200,000×g pellet) consistent with prominent intracellular distribution (Fig. 1B).

To confirm the specificity of the revealed bands, cell extracts from cells transiently transfected with hCaSR-wt, hCaSR-R990G and hCaSR-N124K were probed with anti-GFP antibody. Anti-GFP antibody stained bands at the same molecular weight as those revealed by anti CaSR antibody thus confirming the specific expression of CaSR variants in HEK-293 cells (Fig. 1C).

### Signaling of Wild Type and Mutant Receptors

**Figure 2:**
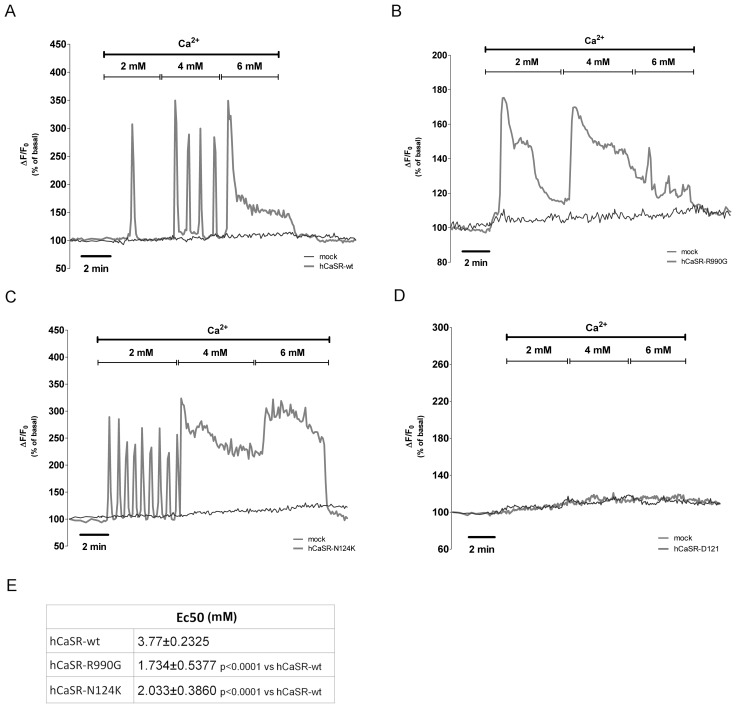
Effects of increasing concentration of extracellular calcium on [Ca^2+^]_i_ levels. Cells transiently transfected with (A) hCaSR-wt, (B) hCaSR-R990G, (C) hCaSR-N124K and (D) hCaSR-Δ121 were stimulated with increasing levels of [Ca^2+^]_o_ and fluorescence ratio 340 nm/380 nm was recorded and calculated as the change in fluorescence (ΔF/F_0_), normalized to basal fluorescence ratio observed in the absence of stimulus (100%). Each trace is representative of 3–4 different experiments with similar results. (E) Calculated EC_50_ for hCaSR-wt, hCaSR-R990G and hCaSR-N124K.

HEK-293 cells were transiently transfected with hCaSR-wt, hCaSR-R990G, hCaSR-N124K and hCaSR-Δ121 and CaSR-stimulated changes in intracellular calcium were measured. Cells were loaded with Fura-2AM (4 µM), exposed to 2 mM, 4 mM and 6 mM extracellular calcium and variations in intracellular calcium (Ca^2+^_i_) were evaluated by single-cell epifluorescence imaging and quantified with respect to basal levels (100%) measuring the maximal change in ΔF/F_0_ (F_0_ is defined by the average of fluorescent intensity at the basal level, whereas ΔF equals to F_1_-F_0_). Figure 2 shows representative time course of fluorescence responses of the wild type and mutant receptors. The HEK-293 cells transfected with hCaSR-wt had a threshold response to extracellular calcium levels of about 2 mM. Activation of hCaSR-wt receptor induced typical calcium oscillations [31] in response to extracellular calcium levels [Ca^2+^]_o_ between 2 mM and 4 mM, while the maximal response of the hCaSR-wt characterized by a sustained intracellular calcium increase, was observed between 5 mM and 6 mM (Fig. 2A; n = 51 cells). In contrast, hCaSR-R990G exhibited a sustained intracellular calcium increase already at 2 mM [Ca^2+^]_o_ consistent with a functional expression of a gain-of-function receptor (Fig. 2B; n = 63 cells). Similarly, hCaSR-N124K showed a greater sensitivity to [Ca^2+^]_o_ displaying calcium oscillations at 2 mM and a sustained intracellular calcium increase starting from 4 mM [Ca^2+^]_o_ (Fig. 2C; n = 56 cells). No changes in intracellular calcium levels were detected in cells expressing the truncated inactive hCaSR-Δ121 (Fig. 2D; n = 38 cells) and in mock cells (n = 70 cells). Calculation of the EC_50_ values obtained by normalization to the maximal response of the wild type receptor, revealed that, compared with hCaSR-wt, both the hCaSR-R990G and the hCaSR-N124K mutant receptors showing a left shifted dose-response curve had significant (P<0.0001) reduced EC_50_ values (Fig. 2E).

**Figure 3:**
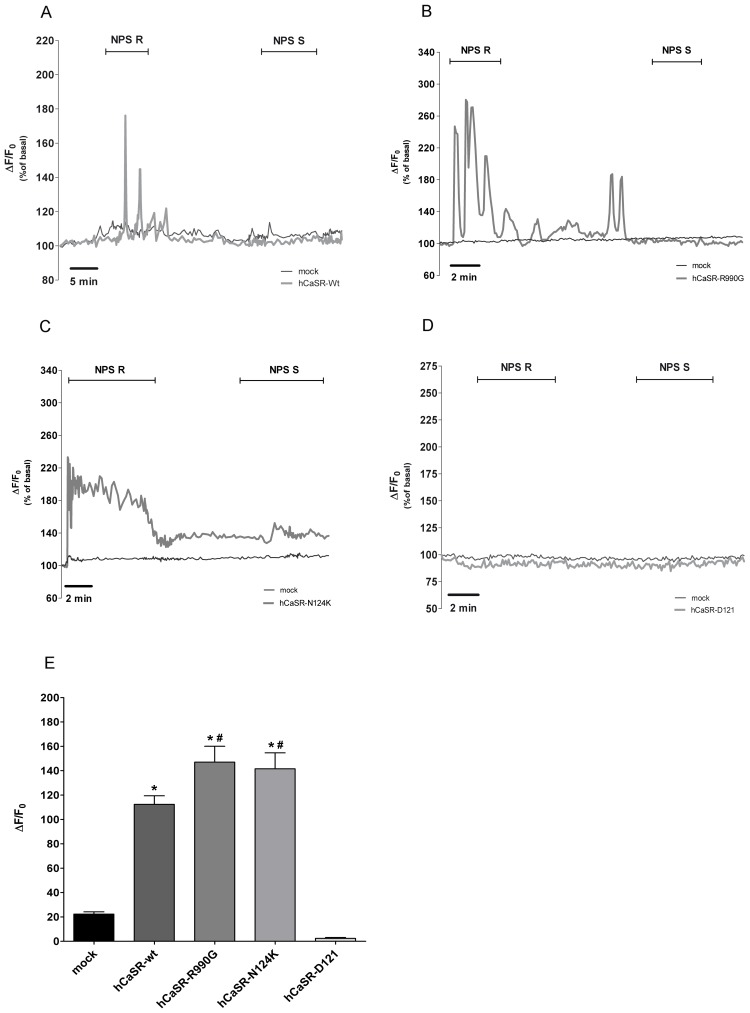
Effects of the calcymimetic NPS-R and its inactive form NPS-S on [Ca^2+^]_i_. Cells transiently transfected with (A) hCaSR-wt, (B) hCaSR-R990G, (C) hCaSR-N124K and (D) hCaSR-Δ121 were stimulated with NPS-R (5 µM) and NPS-S (5 µM). Fluorescence ratio 340 nm/380 nm was recorded and responses to NPS-R were calculated as the change in fluorescence (ΔF/F_0_), normalized to basal fluorescence ratio observed in the absence of stimulus (100%). Each trace is representative of 3 different experiments with similar results. (E). Histogram showing the ΔF/F_0_. Data are expressed as means ± SE. (*P<0.0001 vs mock; #P<0.001 vs hCaSR-wt).

For a more specific functional analysis of wild type and mutant receptors, cells were treated with NPS-R, the allosteric CaSR modulator that increases the sensitivity of the receptor for calcium. A significant increase in intracellular calcium was observed in cells expressing hCaSR-wt (Fig. 3A; n = 27 cells; 112.4±7.05%; *P<0.0001) and its activating variants (Fig. 3B, n = 20 cells, 147.0±13.02%, *P<0.0001; Fig. 3C, n = 21 cells, 141.6±13.08%, *P<0.0001) respect to mock (n = 33 cells; 22.33±1.86%). No response was observed in hCaSR-Δ121 expressing cells (Fig. 3D; n = 25 cells). NPS-S, the much less potent NPS-R enantiomer, was ineffective in all experimental conditions confirming the high specificity of NPS-R effect (Fig. 3A–D). Interestingly, statistical analysis of the fluorescence responses revealed that, compared to the wild type, the activation of two gain-of-function receptors resulted in a significant higher increase in fluorescence response reflecting concomitant significant higher increase in intracellular calcium in response to NPS-R (Fig. 3E).

### Baseline Intracellular Calcium Measurements in Cells Expressing CaSR Variants

Intracellular calcium level is strictly controlled to prevent over-activation of cellular responses and the consequent cytotoxicity due to prolonged exposure to high cytosolic calcium. Therefore, the calcium gradient between cytosol and endoplasmic reticulum (ER) is fundamental for correct transmission of transduction signals.

**Figure 4:**
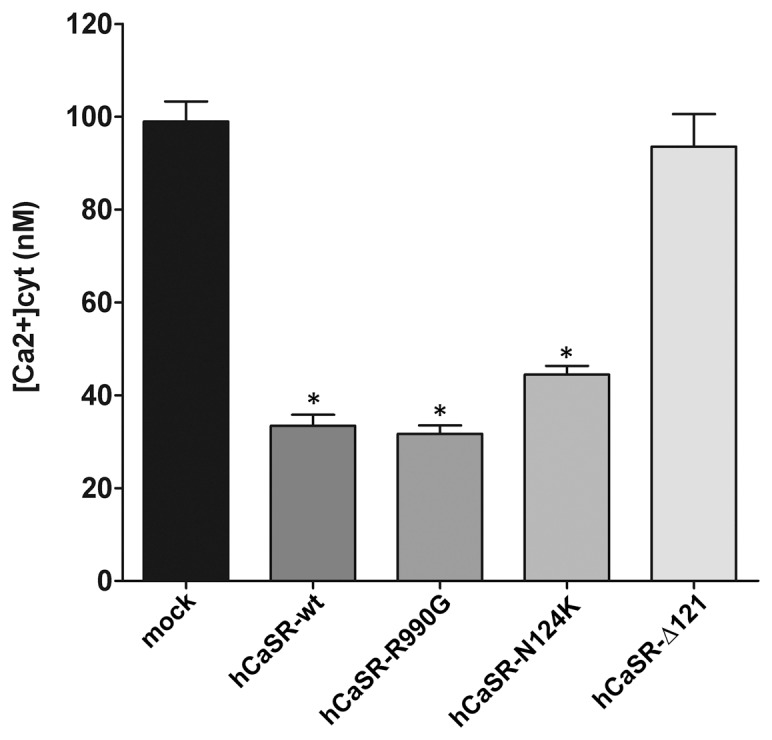
Effect of expression of hCaSR-wt and its variants on free cytosolic [Ca^2+^] at steady state. HEK-293 cells were transiently transfected and the free cytosolic [Ca^2+^] was calculated 48–72 hours after transfection accordingly to Grynkiewicz formula [32], as described in Methods. Cytosolic calcium was measured in mock cells (n = 114 cells), hCaSR-wt (n = 64 cells), hCaSR-R990G (n = 89 cells), hCaSR-N124K (n = 72 cells) and hCaSR-Δ121 (n = 96 cells). Data are expressed as means ± SE. (*P<0.0001 vs mock or hCaSR-Δ121).

We therefore analyzed whether expression of CaSR alters the basal cytosolic calcium. At steady state, cells expressing hCaSR-wt and its gain-of-function variants showed a significant decrease in cytosolic calcium levels compared with mock and hCaSR-Δ121 expressing cells (intracellular calcium concentrations in transfected cells: hCaSR-wt: 33.44±2.39 nM; hCaSR-R990G: 31.67±1.82 nM; hCaSR-N124K: 44.45±1.89 nM; mock: 98.96±4.32 nM; hCaSR-Δ121: 93.58±7.01 nM, *P<0.0001) (Fig. 4). These interesting data indicate that CaSR expression results in modulation of unknown signaling components leading to a significant reduction in intracellular calcium at rest. This is expected to increase cell sensitivity to extracellular signals acting through calcium signaling.

A counterpart measure to increase cell sensitivity to extracellular signals in terms of intracellular calcium release would be to raise the concentration of calcium stored in the ER. This is also expected to increase the ER to cytosol gradient likely resulting in a more pronounced cellular response to CaSR activators.

**Figure 5:**
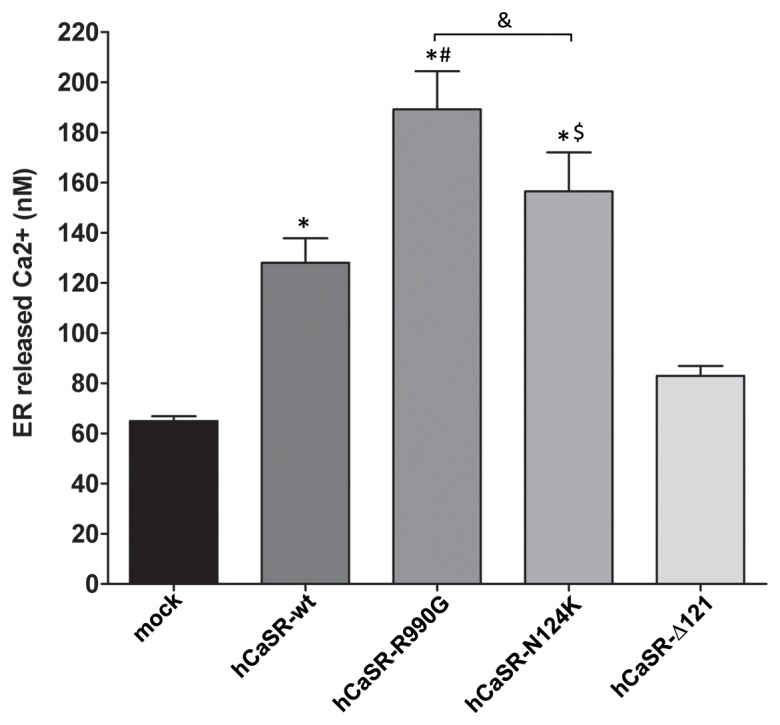
Effect of expression of hCaSR-wt and its variants on calcium release from the internal stores. HEK-293 cells were transiently transfected as described in Methods and the experiments were executed 48–72 hours after transfection. The calcium released by the internal stores was calibrated by adding ionomycin (5 µM) in the absence of Ca^2+^_o_, according to the Grynkiewicz formula [32]. Measurements were performed in mock cells (n = 69 cells), hCaSR-wt (n = 46 cells), hCaSR-R990G (n = 49 cells), hCaSR-N124K (n = 48 cells) and hCaSR-Δ121 (n = 56 cells). Data are expressed as means ± SE. (*P<0.0001 vs mock; #P<0.0001 hCaSR-R990G vs hCaSR-wt; $P<0.001 hCaSR-N124K vs hCaSR-wt; &P<0.01 hCaSR-R990G vs hCaSR-N124K).

In line with this concept, we observed that the bulk of the ER-released calcium was significantly higher in cells expressing hCaSR-wt (128±9.8 nM) and the activating variants (hCaSR-R990G: 189.2±15.24 nM; hCaSR-N124K: 156.5±15.52 nM) compared with mock (50.78±1.85 nM, *P<0.0001) or cells expressing hCaSR-Δ121 (82.99±3.95 nM) (Fig. 5). The experimental strategy employed to test this aspect was to treat cells with ionomycin in the absence of Ca^2+^_o_ [32] (see Methods for details). Under such conditions, ionomycin caused Ca^2+^ release from intracellular stores, resulting in a detectable rise in cytosolic calcium levels**.** Of note, both activating CaSR variants were able to promote significantly higher calcium release from the ER than hCaSR-wt expressing cells (hCaSR-R990G vs hCaSR-wt, #P<0.0001; hCaSR-N124K vs hCaSR-wt, $P<0.01). Indeed, the hCaSR-R990G receptor variant apparently was significantly more efficient at releasing calcium from the ER (hCaSR-R990G vs hCaSR-N124K, &P<0.01).

**Figure 6:**
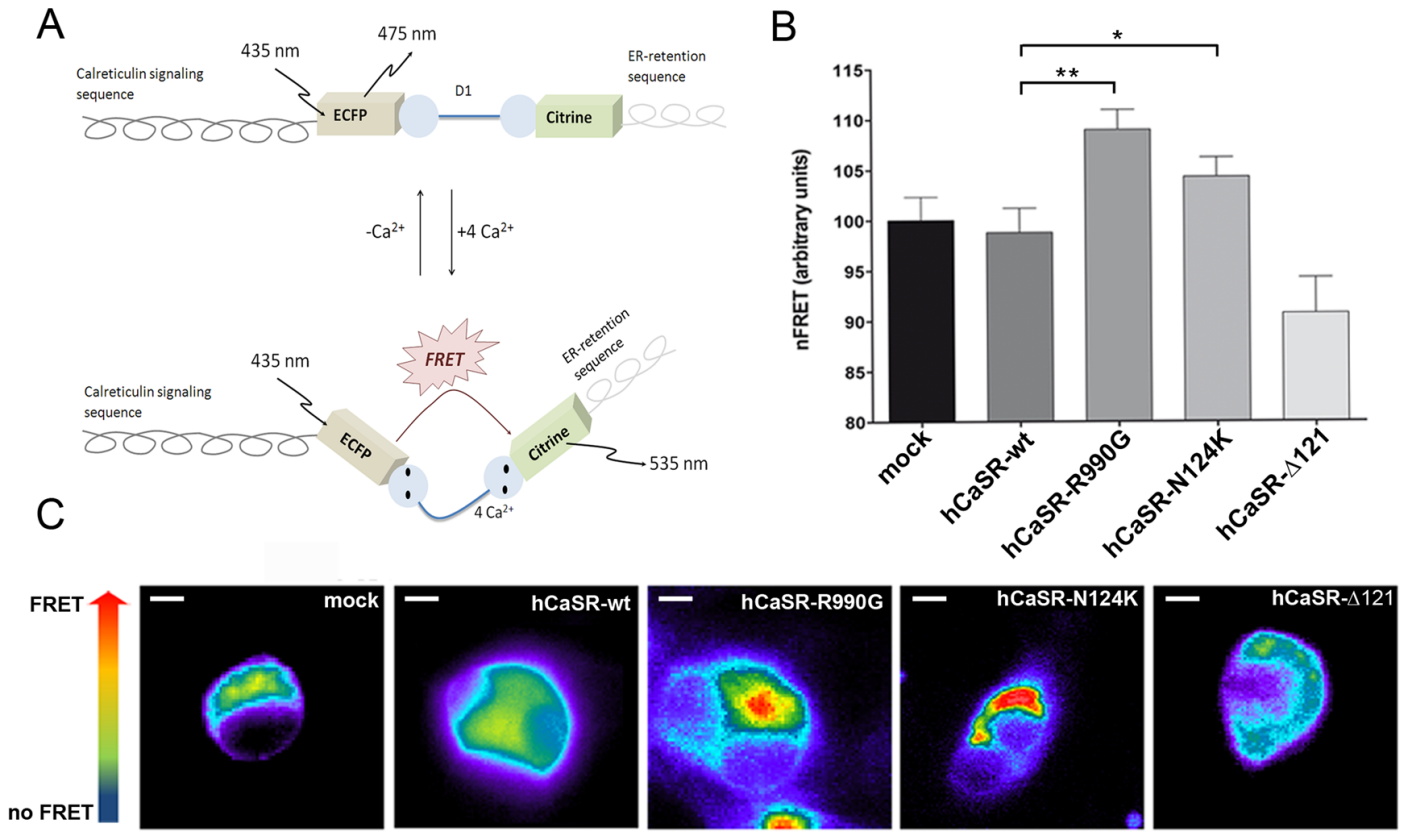
Evaluation of [Ca^2+^]_ER_ with ER-targeted cameleon (D1ER) probe. Schematic model of the cameleon (D1ER) probe showing calreticulin signaling sequence flanked by the ECFP, calcium binding calmodulin domain (D1) and citrine to monitor the ER-calcium stored. Binding of calcium to calmodulin sequence results in an intramolecular rearrangement of the probe consistently with an increase in FRET signal. (B) Histogram compares changes in nFRET among mock cells (n = 98 cells), hCaSR-wt (n = 75 cells), hCaSR-R990G (n = 98 cells), hCaSR-N124K (n = 77 cells) and hCaSR-Δ121 (n = 75 cells). Data are expressed as means ± SE. (**P<0.001; *P<0.01). (C) The FRET signal (ratio of 530/470 nm) is depicted in pseudocolor. The scale bar corresponds to 5 µm.

The next step was to evaluate the actual calcium concentration in the ER. To this end, we performed FRET experiments using the ER-targeted Cameleon (D1ER) probe [33] containing a ER-retention motif, which detects [Ca^2+^]_ER_ directly. A scheme of D1ER is shown in Figure 6A: when calcium binds to the calmodulin motif (D1), it causes an intramolecular rearrangement of the probe which leads to energy transfer between the donor and acceptor molecules, resulting in FRET signal output.

Interestingly, we found that cells expressing the gain-of-function CaSR variants, hCaSR-R990G (112.4±2.04% vs hCaSR-wt 100±1.78%, **P<0.001) and hCaSR-N124K (109.6±1.84% vs hCaSR-wt 100±1.78%, *P<0.01), had a significantly higher calcium concentration in the ER (Fig. 6B). Representative cells expressing CaSR and its variants are indicated in the lower panel where the FRET ratio is depicted in pseudocolor (Fig. 6C).

**Figure 7:**
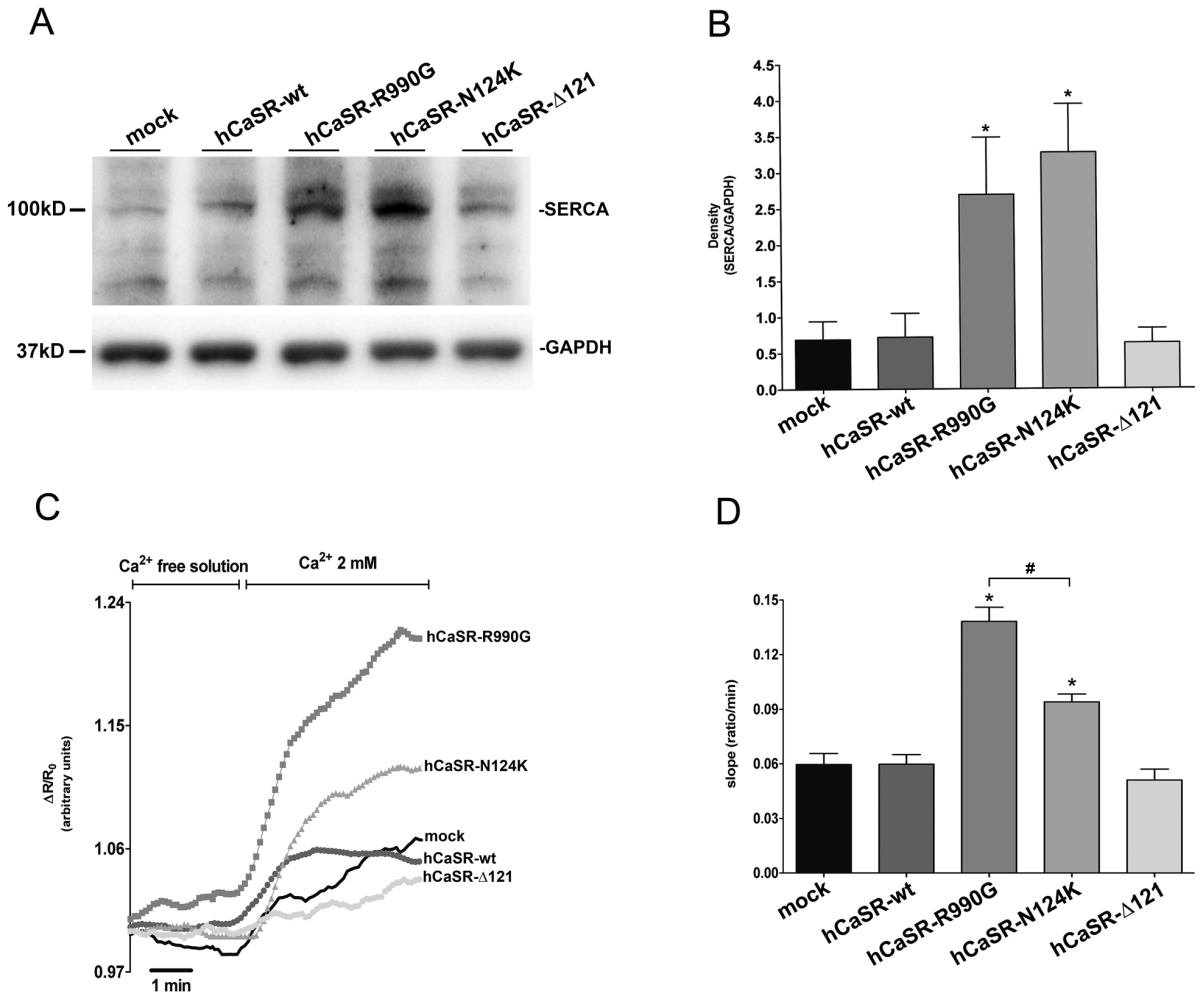
Evaluation of SERCA expression and activity. Western blotting analysis. Equal amount of proteins (60 µg) from cell lysates were immunoblotted and probed with goat polyclonal antibody against SERCA2 (recognizing both the a and b isoforms; 1∶500). (B) Densitometry of the immunoreactive bands. Statistical analysis revealed that the SERCA2 expression, normalized against GAPDH, was significantly higher in cells expressing activating CaSR variants (data expressed as means ± SE; *P<0.01 vs mock or hCaSR-wt). (C) Dynamic FRET. Calcium was depleted from the ER and the speed of calcium re-uptake into the ER was measured with a FRET-based D1ER sensor. (D) Statistical analysis of the SERCA evaluated based on the initial slope of the ratio increase after calcium readdition. Data are expressed as means ± SE. (*P<0.0001 vs hCaSR-wt; #P<0.0001 hCaSR-R990G vs hCaSR-N124K).

The efficiency of calcium accumulation in the ER is strictly dependent on SERCA activity. Therefore, the next step was to evaluate the expression and the activity of SERCA by immunoblotting and dynamic FRET respectively. Cells were lysed and equal amount of proteins (60 µg) were immunoblotted and probed with goat polyclonal antibody against SERCA2 (Fig. 7A). Statistical analysis revealed that SERCA expression was significantly higher in cells expressing activating CaSR variants (hCaSR-R990G: 2.69±0.79; hCaSR-N124K: 3.27±0.67) compared to mock (0.69±0.25, *P<0.01), hCaSR-wt (0.72±0.33, *P<0.01) and hCaSR-Δ121 (0.63±0.20, *P<0.01) expressing cells (Fig. 7B). Of note, dynamic FRET revealed that the increase in SERCA abundance is accompanied by a parallel increase in its activity. The experimental strategy was to deplete calcium from the ER using cyclopiazonic acid (CPA) and to measure the speed of calcium re-uptake into the ER using a FRET-based D1ER sensor (Fig. 7C). hCaSR-R990G and hCaSR-N124K expressing cells showed a significant increased ability to accumulate calcium in the ER via SERCA respect to hCaSR-wt (*P<0.0001), as indicated by the quantification of the initial slope of the FRET ratio (mock: 0.06±0.006; hCaSR-wt: 0.06±0.005; hCaSR-R990G: 0.138±0.008; hCaSR-N124K: 0.094±0.004; hCaSR-Δ121: 0.05±0.006 ratio/min, Fig. 7C–D). Indeed, hCaSR-R990G expressing cells displayed significantly higher SERCA activity compared to the hCaSR-N124K variant (#P<0.0001).

The emerging picture is that a key point in regulating the efficiency of CaSR signaling is to increase the ER to cytosol gradient. For this, cells appear to monitor cytosolic and ER Ca^2+^ levels by modulating the expression of key signaling proteins. We next evaluated whether cells also adjust the expression of the PMCA pump and calbindin in order to stabilize the system.

**Figure 8:**
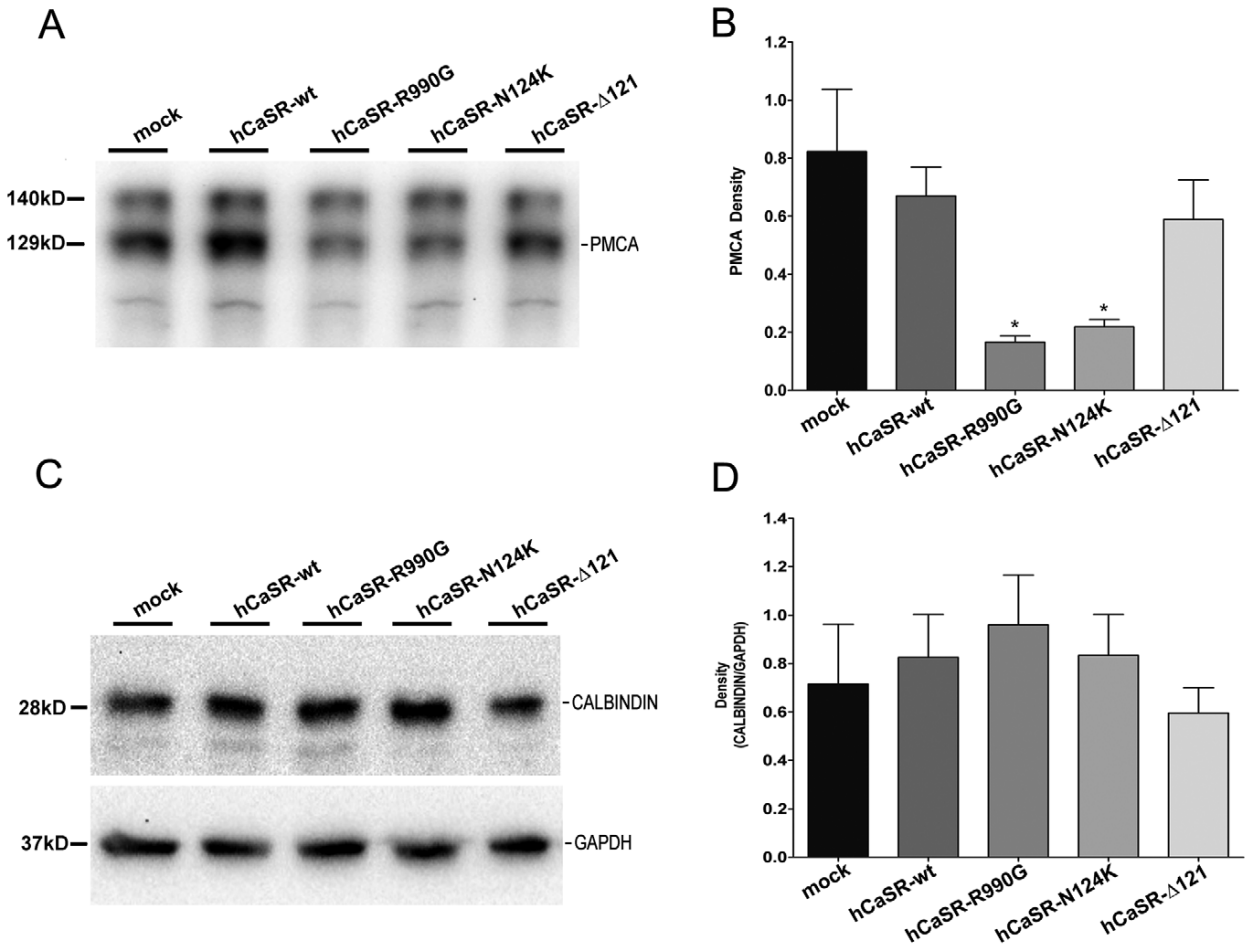
PMCA and Calbindin expression. (A) Western blotting analysis of PMCA. Fractions enriched in plasma membrane were prepared and equal amounts of proteins (60 µg) from each cell line were subjected to Western Blotting analysis with anti-PMCA (1∶300) and revealed with anti-mouse HRP-coupled secondary antibodies. (B) Statistical analysis of the detected bands. PMCA abundance was significantly reduced in cells expressing activating CaSR variants (means ± SE; *P<0.01 vs hCaSR-wt or mock). (C) Western blotting analysis of Calbindin. Equal amounts of proteins (60 µg) from cell lysates were immunoblotted, probed with monoclonal antibodies against calbindin (N-terminal, 1∶2000) and revealed with anti-mouse HRP-coupled secondary antibodies. (D) Densitometric analysis of the revealed bands. No significant change in calbindin abundance was observed in cells expressing hCaSR-wt or its activating and inactivating variants.

Cell lysates (60 µg) were subjected to immunoblotting and probed with specific antibodies recognizing PMCA1/4 (Fig. 8A). Results revealed that the expression level of PMCA was significantly reduced in hCaSR-R990G (0.166±0.02) and hCaSR-N124K (0.219±0.03) cells (Fig. 8B) compared to that observed in mock (0.823±0.22, *P<0.01), hCaSR-wt (0.670±0.09, *P<0.01) and hCaSR-Δ121 (0.588±0.14, *P<0.01) expressing cells. These results are in agreement with the comparable reduction in baseline calcium concentration measured in hCaSR-wt, hCaSR-R990G and hCaSR-N124K expressing cells. A reduction in PMCA expression observed only in activating CaSR variants expressing cells might explain why those cells, at rest, display similar calcium concentrations compared with hCaSR-wt expressing cells.

No significant changes in the protein abundance of calbindin were detected (Fig. 8C–D).

## Discussion

In this study, we demonstrated for the first time that functional expression of gain-of-function variants of the GPCR CaSR results in parallel modulation of the expression level and activity of an integrated set of regulatory proteins contributing to an excessive signal transduction of mutant receptors.

Specifically the main findings reported in this work are summarized as follows: (*i)* both the hCaSR-R990G and the hCaSR-N124K mutant receptors had significant reduced EC_50_ values when compared with hCaSR-wt, (*ii)* basal intracellular calcium concentration was significantly lower in cells expressing hCaSR-wt and its activating variants compared to mock or loss-of-function hCaSR-Δ121 expressing cells, (*iii*) FRET experiments with the D1ER probe, which detects [Ca^2+^]_ER_ directly, demonstrated a significantly higher calcium accumulation in the ER in cells expressing the activating CaSR variants compared to hCaSR-wt expressing cells, (*iv*) SERCA expression and activity increased significantly in cells expressing activating CaSR variants compared to hCaSR-wt expressing cells. An inverse correlation with PMCA was also found.

All these findings demonstrate that cells ‘sense’ expression of the CaSR and adopt multiple parallel adaptive regulatory mechanisms aimed at controlling the expression levels of crucial regulatory proteins involved in calcium homeostasis. This combined integrated protein regulation on the one hand contributes to lowering resting intracellular calcium concentration and on the other increases calcium accumulation in the ER.

Stabilization of basal cytosolic calcium to low levels (50 nM range) and the differential higher accumulation of calcium in the ER due to higher SERCA activity in cells expressing CaSR activating variants, are predicted to render cells more sensitive to extracellular CaSR agonists. Of note we found that cells adjust the expression levels of PMCA on the plasma membrane, which was reduced in cells expressing the gain-of-function CaSR variants. This likely implies that comparable levels of intracellular resting calcium are reached either in cells expressing hCaSR-wt or activating CaSR variants. While non-selective plasma membrane calcium channels could also contribute to the low basal level of cytosolic calcium observed in cells expressing functional CaSR variants, our findings point to SERCA and PMCA as two candidate transporters involved in this effect.

Altogether, our data provide significant new knowledge to help explain the molecular basis of gain-of-function variants of the calcium-sensing receptor (CaSR). In fact, while previous studies showed that the functional consequence of expressing gain-of-function mutations of the CaSR is basically higher sensitivity to external calcium as assessed by reduced EC_50_ values for external calcium [23], the present contribution indicates that variations in key protein expression explain how the calcium signaling system is affected (and exacerbated) by activating CaSR variants.

As previously discussed, the gain-of-function CaSR mutations result in autosomal dominant hypocalcemia (ADH) or type 5 Bartter syndrome and eight of them are clustered in loop 2 close to the two cysteines responsible for receptor homodimerization, i.e. cys 129 and cys 131 [34]. The missense mutation N124K analyzed in this work is located in the CaSR extracellular domain in loop 2 and this substitution has been previously shown to cause an increase in sensitivity to external calcium rather than cause constitutive activation [20]. Based on the three-dimensional structure of related metabotropic glutamate type 1 receptor, it has been speculated that the N124K mutation of CaSR changes the receptor structure, decreasing the normal constraints to rotation of the dimer [20]. On the other hand, a gain-of-function effect is also observed in autoimmune polyglandular syndrome type 1, in which autoantibodies bind the N-terminus of the CaSR stabilizing activating conformation of the receptor [35].

While the N124K CaSR mutations causing ADH in humans is located in the extracellular domain, the intracellular tail of the receptor has one non-conservative polymorphism, R990G, which also confers a gain-of-function to the receptor (lower external calcium EC_50_) and in humans is associated with primary hypercalciuria in patients [19], [21], [36].

Although the two gain-of-function CaSR variants examined in this work regard modifications at opposite locations within the CaSR protein sequence, their functional analysis revealed comparable biological regulatory effects within cells: a. significantly higher calcium accumulation in the ER and SERCA expression and activity, b. reduced expression of the PMCA, which is perfectly in line with the comparable (low) basal cytosolic calcium concentration found in cells expressing hCaSR-wt.

These results likely reflect the need to preserve optimal intracellular calcium concentrations, while assuring differential responses for hCaSR-wt or activating CaSR variants. In fact, the observed significant higher activity of the SERCA pump might cause a dangerous drop in basal cytosolic calcium which can disrupt the normal activities of calcium dependent proteases, phosphatases and other effectors [37]. To avoid that, the selective downregulation of PMCA observed in activating CaSR variant expressing cells (Fig. 8) represents a very efficient feedback regulation in order to stabilize the system. The relevance of the SERCA-PMCA interplay in maintaining the calcium gradient across cellular membranes is also confirmed by previous studies in SERCA and PMCA knockout mice [28]. Moreover prolonged stimulation of the CaSR signaling might modulate gene expression contributing to modify cellular phenotype [38], [39].

The emerging picture here is that cells respond to the functional expression of the CaSR activating variants with a series of adaptive mechanisms. Our data point to the concept that multiple parallel adaptive feedback loops ‘sense’ ectopic expression of the CaSR variants by altering ER and cytosolic calcium concentrations and adjusting the expression levels of SERCA and PMCA for efficient signaling. In this scenario, Ca^2+^ plays an important role in controlling the expression patterns of calcium regulated players that are differentially modulated in both health and disease [40].

Given the complexity of feedbacks in the calcium signaling system analyzed in this work, it is clearly difficult to understand the mechanisms behind these parallel adaptive regulations of protein expression and activity. Clues in this direction come from an elegant recent work by Gong and coworkers, demonstrating that activation of the CaSR regulates the expression levels of two microRNAs: miR-9 and miR-374, which in turn transduce the extracellular signal to CLDN14 through microRNA mediated gene silencing [41]. Although, this study reported miR-9 and miR-374 convergence onto CLDN14, regulation of microRNA by CaSR signaling may occur on several layers including key regulatory proteins involved in CaSR receptor activated calcium signaling.

In conclusion, our findings identify a unique adaptive principle in the calcium signaling system associated with activating variants of the CaSR whereby SERCA, PMCA and possibly other signaling components are regulated by multiple expression feedbacks so that modifications in ER and cytosolic calcium levels are paralleled by modified concentrations of key signaling pathway components. This combined parallel regulation in protein expression increases the ER to cytosol calcium gradient explaining the higher sensitivity of CaSR gain-of-function variants to external calcium.

## Materials and Methods

### Materials

All chemicals were purchased from Sigma (Sigma-Aldrich, Milan, Italy). Fura-2AM was obtained from Molecular Probes (Life Technologies, Monza, Italy). NPS-R568 and the enantiomer NPS-S568 were kindly gifted by Amgen (Amgen Dompé S.p.a., Milan, Italy).

### Antibodies

Monoclonal CaSR antibody recognizing amino-acid 15–29 at the extracellular N-terminus [42], [43] was from Sigma-Aldrich, Milan, Italy. Calbindin-28K antibody was purchased from Sigma whereas PMCA1/4 and SERCA2 (N-19 recognizing both the a and b isoform) antibodies were obtained from Santa Cruz Biotechnologies (Tebu Bio, Milan, Italy). Monoclonal antibody against Green Fluorescent Protein (GFP) was from Covance (Emeryville, CA, USA). Monoclonal antibody against Glyceraldehyde-3-Phosphate Dehydrogenase (GAPDH, clone 6C5) was purchased from Millipore (Millipore Corporation, USA).

### DNA Constructs

For generation of the PCR3.1 constructs encoding for human CaSR wild-type (hCaSR-wt) and its variants (hCaSR-R990G; hCaSR-N124K; hCaSR-Δ121, the coding sequences were amplified by PCR and subcloned in frame in pAC-GFP1-N1. The obtained GFP-fused mutants are functional and correctly targeted to the plasma membrane. The presence of the GFP tag does not alter CaSR function and trafficking as previously described [44].

### Cell Culture and Transfection

Human embryonic kidney (HEK-293) cells, were grown in Dulbecco’s Modified Eagle’s Medium (DMEM) high glucose, GlutaMAX™, pyruvate, supplemented with 10% (v/v) fetal bovine serum and 100 i.u./ml penicillin, 100 µg/ml streptomycin at 37°C in 5% CO_2_. HEK-293 cells were seeded on a poly-L-lysine hydrobromide substrate and grown for 24 h at 80% confluence.

Cells were transiently transfected with plasmids (0.4 µg of DNA/cm^2^) encoding for hCaSR-wt and its variants fused with the green fluorescent protein (GFP), using lipofectamine (1 µg/µl) according to the protocol provided by the manufacturer (Life Technologies, Monza Italy). Experiments were performed 48–72 hours post-transfection.

### Cell Preparations

HEK-293 cells were lysed in RIPA Buffer (150 mM NaCl, 10 mM Tris, pH 7.2, 0.1% SDS, 1.0% Triton X-100, 1% Deoxycholate, 5 mM EDTA) in the presence of proteases (1 mM PMSF, 2 mg/ml leupeptin and 2 mg/ml pepstatin A) and phosphatases (10 mM NaF and 1 mM sodium orthovanadate) inhibitors. Cellular debris was removed by centrifugation at 12,000×g for 20 min at 4°C. The supernatants were collected and used for immunoblotting studies. Alternatively, cells were scraped and sonicated in a buffer containing 220 mM mannitol, 70 mM sucrose, 20 mM Tris-HCl in the presence of protease and phosphatase inhibitors. Nuclei and mitochondria enriched fractions were removed by centrifugation at 800×g and 8000×g respectively for 20 minutes at 4°C. Membrane enriched fractions were obtained by centrifugation for 1 hour at 4°C at 17,000×g in a Beckman Allegra 64R centrifuge. The supernatants were spun at 200,000×g for 1 hour at 4°C.

### Gel Electrophoresis and Immunoblotting

Cellular proteins were separated on 10% bis-tris acrylamide gels under reducing conditions. Protein bands were electrophoretically transferred onto Immobilon-P membranes (Millipore Corporate Headquarters, Billerica, USA) for Western blot analysis, blocked in TBS-Tween-20 containing 3% BSA and incubated with primary antibodies O/N.

Immunoreactive bands were detected with secondary antibody conjugated to horseradish peroxidase (HRP) obtained from Santa Cruz Biotechnologies (Tebu Bio, Milan, Italy). Membranes were developed using SuperSignal West Pico Chemiluminescent Substrate (Pierce, Rockford, USA) with Chemidoc System (Bio-Rad Laboratories, Milan, Italy). Band intensities were quantified by densitometric analysis using National Institutes of Health (NIH) ImageJ software.

### CaSR Cellular Localization

HEK-293 cells were seeded onto Ø10 mm glass coverslips, grown, transfected as described above, and fixed for 30 min with 4% paraformaldehyde in PBS. Following fixation, samples were mounted on glass slides with Mowiol. Images were obtained with a confocal microscope Leica TCS SP2 (Leica Microsystems, Heerbrugg, Switzerland).

### Functional Analysis of Wild-Type and Mutant Receptors. Intracellular Calcium Measurements

For intracellular Ca^2+^ measurements, cells were grown on Ø40 mm glass coverslips. HEK-293 cells were loaded with 4 µM Fura-2AM for 20 min at 37°C in DMEM. Ringer’s Solution was used to perfuse cells during the experiment containing 140 mM NaCl, 5 mM KCl, 1 mM MgCl_2_, 10 mM Hepes, 5 mM Glucose, 1.8 mM CaCl_2_, pH 7.4. In fluorescence measurements, the coverslips with dye-loaded cells were mounted in a perfusion chamber (FCS2 Closed Chamber System, BIOPTECHS, Butler, U.S.A.) and measurements were performed using an inverted microscope (Nikon Eclipse TE2000-S microscope) equipped for single cell fluorescence measurements and imaging analysis. The sample was illuminated through a 40X oil immersion objective (NA = 1.30). The Fura-2AM loaded sample was excited at 340 and 380 nm. Emitted fluorescence was passed through a dichroic mirror, filtered at 510 nm (Omega Optical, Brattleboro, VT, USA) and captured by a cooled CCD camera (CoolSNAP HQ, Photometrics). Fluorescence measurements were carried out using Metafluor software (Molecular Devices, MDS Analytical Technologies, Toronto, Canada). The ratio of fluorescence intensities at 340 and 380 nm was plotted and calculated as the change in fluorescence (ΔF/F_0_), expressed as percentage of the basal fluorescence ratio observed in the absence of stimulus.

The Ec_50_ shift was calculated by normalization to the maximal response of the wild type receptor and obtained with Graph Prism program.

For the experiments at steady state, intracellular calcium level was calibrated and then calculated as described by Grynkiewicz [32]. Briefly, calcium concentration was determined from the emission fluorescence ratio of the two excitation wavelengths accordingly to the formula [Ca^2+^]_i_ = K_d_*Q(R-Rmin)/(Rmax-R), where K_d_ (224 nM) indicated the dissociation constant of Fura-2AM for Ca_i_ and Q indicated the ratio of the fluorescence intensities (F) at the minimum and the maximum calcium concentration at 380 nm. Each sample was calibrated by the addition of 5 µM ionomycin in presence of 1 mM EGTA (Rmin) followed by 5 µM ionomycin in 5 mM CaCl_2_ (Rmax).

### Fluorescence Resonance Energy Transfer (FRET) Measurements

FRET experiments were performed as described [45]. Briefly, HEK-293 cells were transiently co-transfected with plasmids encoding CaSR-wt or its variants and D1ER cameleon, (gift from Prof. Roger Tsien) [33], for FRET studies. FRET measurements were carried out using MetaMorph software (Molecular Devices, MDS Analytical Technologies, Toronto, Canada). ECFP and citrine were excited at 435 or 509 nm, respectively. FRET from ECFP to citrine was determined by excitation of ECFP and measurement of fluorescence emitted from citrine. Corrected nFRET values were determined accordingly to Tamma et al. [45].

### Statistical Analysis

Data are reported as mean values ± SEM. Statistical analysis was performed by one-way ANOVA followed by Newman-Keuls Multiple Comparison test with p<0.05 were considered statistically different.
